# Intracranial aneurysm circulating exosome-derived LncRNA ATP1A1-AS1 promotes smooth muscle cells phenotype switching and apoptosis

**DOI:** 10.18632/aging.205821

**Published:** 2024-05-08

**Authors:** Chao Wang, Hong Li, Han Zhou, Yifan Xu, Shifang Li, Meng Zhu, Bing Yu, Yugong Feng

**Affiliations:** 1 Department of Neurosurgery, The Affiliated Hospital of Qingdao University, Qingdao, People’s Republic of China; 2 Clinical Laboratory, Central Laboratory, Qingdao Hiser Hospital Affiliated of Qingdao University (Qingdao Traditional Chinese Medicine Hospital), Qingdao, People’s Republic of China; 3 Department of Ophthalmology, The Affiliated Hospital of Qingdao University, Qingdao, People’s Republic of China

**Keywords:** intracranial aneurysm, exosome, LncRNA, phenotype switching, apoptosis

## Abstract

Exosomal long non-coding RNAs (LncRNAs) play a crucial role in the pathogenesis of cerebrovascular diseases. However, the expression profiles and functional significance of exosomal LncRNAs in intracranial aneurysms (IAs) remain poorly understood. Through high-throughput sequencing, we identified 1303 differentially expressed LncRNAs in the plasma exosomes of patients with IAs and healthy controls. Quantitative real-time polymerase chain reaction (qRT-PCR) verification confirmed the differential expression of LncRNAs, the majority of which aligned with the sequencing results. ATP1A1-AS1 showed the most significant upregulation in the disease group. Importantly, subsequent *in vitro* experiments validated that ATP1A1-AS1 overexpression induced a phenotype switching in vascular smooth muscle cells, along with promoting apoptosis and upregulating MMP-9 expression, potentially contributing to IAs formation. Furthermore, expanded-sample validation affirmed the high diagnostic value of ATP1A1-AS1. These findings suggest that ATP1A1-AS1 is a potential therapeutic target for inhibiting IAs progression and serves as a valuable clinical diagnostic marker.

## INTRODUCTION

Intracranial aneurysms (IAs) are focal dilations of the intracranial arterial wall primarily located within the circle of Willis. It is a prevalent cerebrovascular disorder affecting approximately 3.2% of the general population [1]. The annual risk of cerebral aneurysm rupture is approximately 1%. It causes subarachnoid haemorrhage (SAH), resulting in a mortality rate of up to 40% and a disability rate of up to 50%, severely affecting the quality of life [2]. Currently, the main treatment methods for IAs are craniotomy clipping and endovascular intervention [3]. The absence of a safe and effective non-invasive treatment is mainly due to the insufficient understanding of the mechanisms underlying the formation, progression, and rupture of IAs. Furthermore, IA diagnosis relies heavily on invasive angiographic techniques, including computed tomography angiography (CTA) and digital subtraction angiography (DSA), which are unsuitable for large-scale population disease screening [4]. Therefore, investigation of the pathophysiological mechanisms underlying the development and pathogenesis of IAs to identify potential therapeutic targets and biomarkers is crucial.

Exosomes, which are characterised by their lipid bilayer structures, are small vesicles secreted by a variety of cells that serve as pivotal mediators of intercellular communication [5]. They are 30–150 nm in diameter and are found in various body fluids [6]. They contain a range of biomolecules, including DNA, protein fragments, and non-coding RNAs (ncRNAs) [7]. Exosomes play a crucial role in mediating intercellular communication and in the progression of various diseases, including immune responses, cardiovascular diseases, neurological disorders, and cancers [6]. ncRNAs are a class of RNA molecules that lack the potential to encode proteins, widely present in tissues and cells, and participate in the regulation of gene expression through various mechanisms [8, 9]. Research has reported its close association with the pathogenesis of diseases such as cancer, cardiovascular disorders, and neurological conditions [10–12].

Among the ncRNAs, long non-coding RNAs (LncRNAs) have garnered substantial interest in recent years. LncRNAs are transcripts exceeding 200 nucleotides in length and do not encode proteins [13]. Numerous studies have revealed their crucial roles in the development of various human diseases [14–16]. In the context of IAs, LncRNAs play a role in processes such as vascular smooth muscle cells (VSMCs) phenotype switching, cell necrosis and apoptosis, inflammatory activation, endothelial dysfunction, and extracellular matrix disruption, ultimately contributing to aneurysm formation or rupture [17–19]. Certain LncRNAs in the blood of patients with IA were significantly different from those in control subjects, indicating their potential use as diagnostic indicators for IAs [20]. A major challenge in using LncRNAs as clinical diagnostic targets is their instability in plasma, which makes them susceptible to degradation by RNase. However, exosomes protect LncRNAs from degradation, ensuring their stability while crossing physiological barriers [21]. These stable exosomal LncRNAs have advantages over their freely circulating counterparts [22]. They are easier to detect and more accurately reflect LncRNA expression in various diseases. This has sparked research interest in the use of exosomal LncRNAs as diagnostic tools for conditions such as IAs, exploiting the protective properties of exosomes for more reliable clinical detection.

Exosomal LncRNAs play a crucial role in the pathomechanisms of cancers, cardiovascular diseases and central nervous diseases; however, their expression and functional implications in IAs remain unexplored [23–27]. In this study, we applied next-generation sequencing (NGS) to profile the differentially expressed LncRNAs in circulating exosomes from IA patients, subsequently confirming these findings through quantitative real-time polymerase chain reaction (qRT-PCR). Notably, ATP1A1-AS1 expression was most significantly upregulated in IA circulating exosomes. Through knockdown and overexpression experiments in VSMCs, we found that ATP1A1-AS1 promotes VSMCs phenotype switching, facilitates MMP9 expression, and promotes apoptosis. Our research findings indicate that ATP1A1-AS1 may play a crucial role in the pathomechanism of IAs, offering potential as a reliable clinical target and diagnostic biomarker.

## RESULTS

### Identification of exosomes

**Figure 1:**
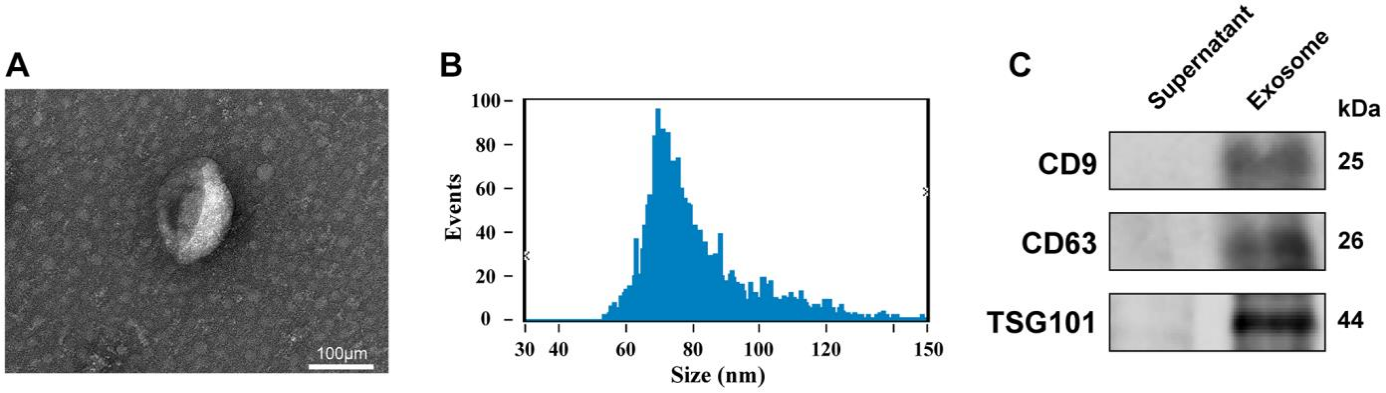
**Identification of exosomes.** (**A**) Transmission electron microscopy images of plasma exosomes (scale bar: 100 μm). (**B**) Size distribution measurements of exosomes by NTA detection. (**C**) Expression level of CD9, CD63, and TSG101 in the isolated exosomes and plasma supernatant, as quantified via WB.

The isolated and purified exosomes were characterised by electron microscopy, nanoparticle tracking analysis (NTA), and western blot (WB). Electron microscopy revealed double-layered spherical vesicles with diameters of 30–150 nm, confirming the presence of exosomes (Figure 1A). We used NTA to evaluate the size distribution of the purified extracellular vesicles, which showed the average diameter of exosomes to be 81.16 nm (Figure 1B). Additionally, WB confirmed the presence of exosomal markers, including CD9, CD63, and TSG101, in the isolated vesicles but not in the plasma supernatant (Figure 1C). Collectively, these comprehensive findings demonstrate the successful isolation and purification of exosomes.

### Circulating exosomal LncRNAs expression and characterisation

**Figure 2:**
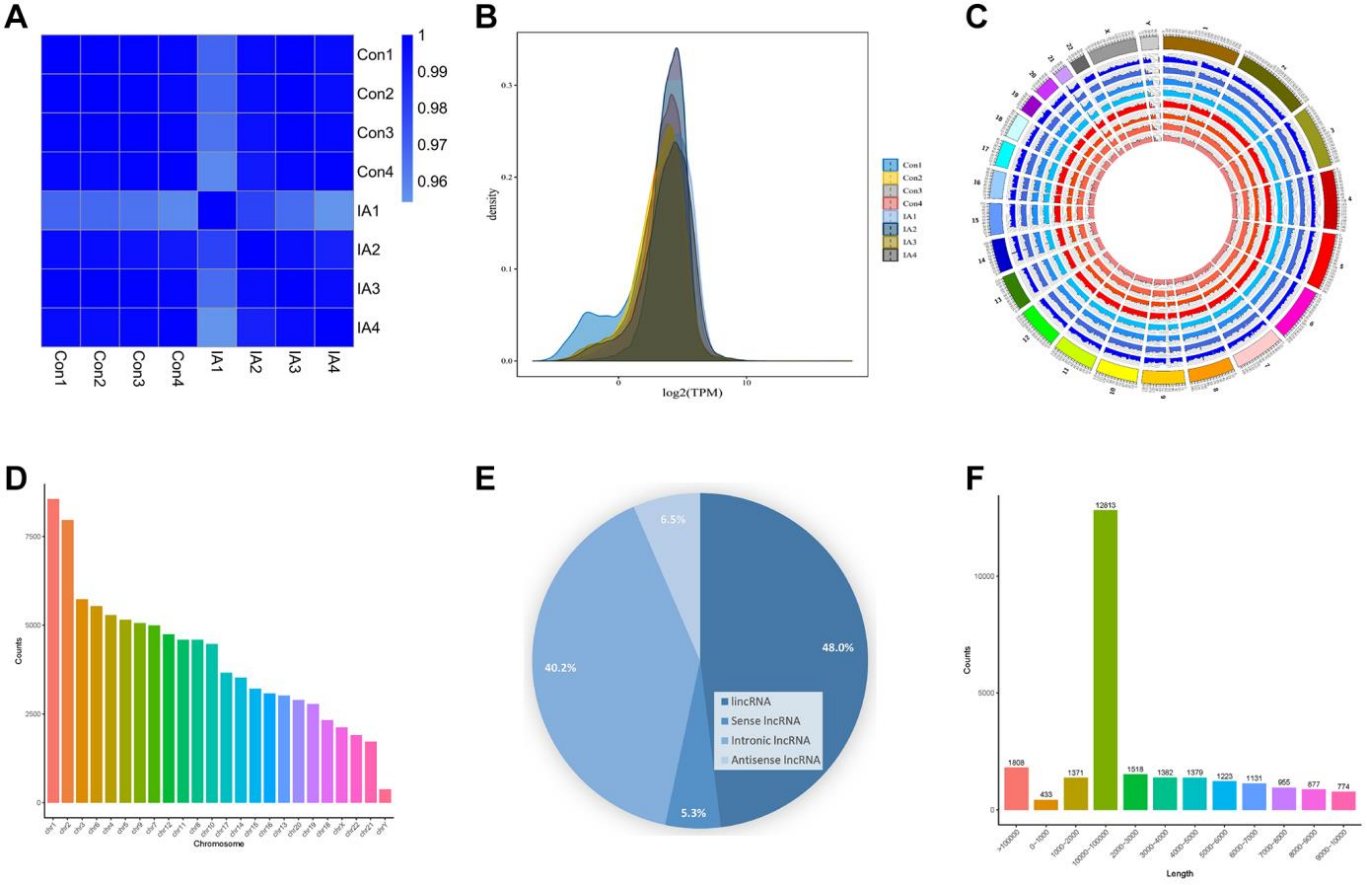
**Circulating exosomal LncRNA expression and characterisation.** (**A**) Heat map of correlation coefficients for all samples. The colours represent the correlation coefficients between pairs of samples. (**B**) Gene expression density map. (**C**) The circular diagram depicting the distribution of LncRNAs on chromosomes illustrates how LncRNAs are distributed on chromosomes across different samples. In the diagram, the outermost layer depicts the genome's chromosome ring, while the inner layer illustrates the distribution of different samples. (**D**) Histogram displaying the distribution of LncRNAs across various chromosomes. (**E**) Pie chart depicting the proportion of different LncRNAs. (**F**) LncRNAs length distribution.

After the purification of exosomal RNA, a library was constructed through reverse transcription synthesis of complementary DNA (cDNA), end repair, splicing, PCR amplification, and other necessary steps. Subsequently, they were sequenced after passing quality control evaluation. The association between samples was quantified using the Pearson correlation coefficient (R) as a metric to gauge the consistency of the obtained outcomes. The R^2^ coefficients for all specimens surpassed 0.9, signifying robust correspondence between duplicate samples and a high degree of correlation in LncRNA expression within the respective groups (Figure 2A). We used density distribution plots based on transcripts per kilobase of exon model per million mapped reads (TPM) to examine the collective gene expression patterns across all samples (Figure 2B). An illustration of LncRNA distribution across individual chromosomes is presented in Figure 2C. These LncRNAs exhibited a uniform distribution pattern across all chromosomes, although a notably larger number of LncRNAs was observed on chromosomes 1 and 2 (Figure 2D). A total of 25,664 LncRNAs were identified using high-throughput sequencing, including 12,324 lincRNAs (48.0%), 1,366 positive-sense LncRNAs (5.3%), 10,312 intronic LncRNAs (40.2%), and 1,662 antisense LncRNAs (6.5%) (Figure 2E). The most prevalent length range was 104–105 nucleotides (nt), with 12,813 LncRNAs falling within this length category (Figure 2F).

### Differential expression (DE) analysis of exo-LncRNAs and functional enrichment analysis

**Figure 3:**
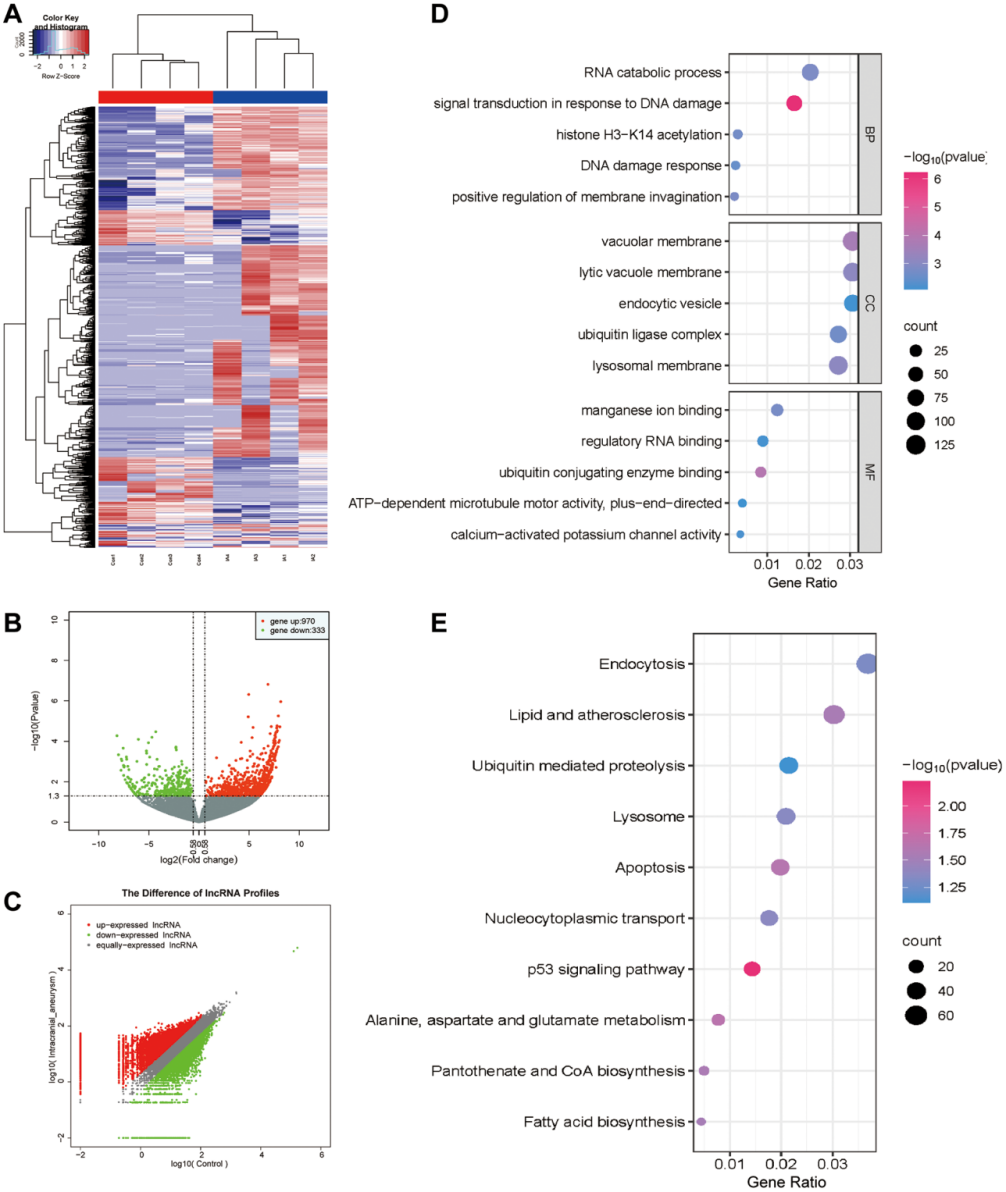
**DE analysis of exo-LncRNAs and functional enrichment analysis.** (**A**) The DE LncRNAs in exosomes obtained from patients with IA and the control group. The upregulated and downregulated LncRNAs are shown in red and blue, respectively. (**B**) Volcano plot of the DE LncRNAs. (**C**) Scatter plot of the DE LncRNAs. (**D**) GO bubble plot of DE LncRNAs target genes. (**E**) KEGG bubble plot of DE LncRNAs target genes.

In this study, we investigated the DE LncRNAs in exosomes derived from patients with aneurysms and healthy controls. Based on the criteria of a fold change exceeding 1.5, and a false discovery rate <0.05, 1303 DE exo-LncRNAs were identified (Figure 3A), including 970 upregulated and 333 downregulated exo-LncRNAs (Figure 3B, 3C). LncRNAs primarily exert their functions by regulating the target genes of the encoded proteins, and their modes of action can be divided into two categories: cis-regulation and trans-regulation. We predicted the target genes of the DE LncRNAs based on the modes of action of LncRNAs and mRNAs, providing a valuable reference for further comprehensive investigations on the functionality of LncRNAs. Functional enrichment analysis revealed that the genes associated with the LncRNA regulatory network were predominantly linked to Gene Ontology (GO) terms, including ‘signal transduction in response to DNA damage’, ‘DNA damage response’, ‘vacuolar membrane’, and ‘endocytic vesicle’ (Figure 3D). Furthermore, to gain deeper insights into the biological functionality, Kyoto Encyclopedia of Genes and Genomes (KEGG) pathway enrichment analysis was performed on the related genes within the LncRNA regulatory network. In summary, these genes were primarily involved in ‘lipid metabolism and atherosclerosis’, ‘apoptosis’, and ‘endocytosis’ (Figure 3E). These results suggest that these DE LncRNAs play various pathophysiological roles and may be involved in the regulation of IAs through these pathways.

### Verification of qRT-PCR

**Figure 4:**
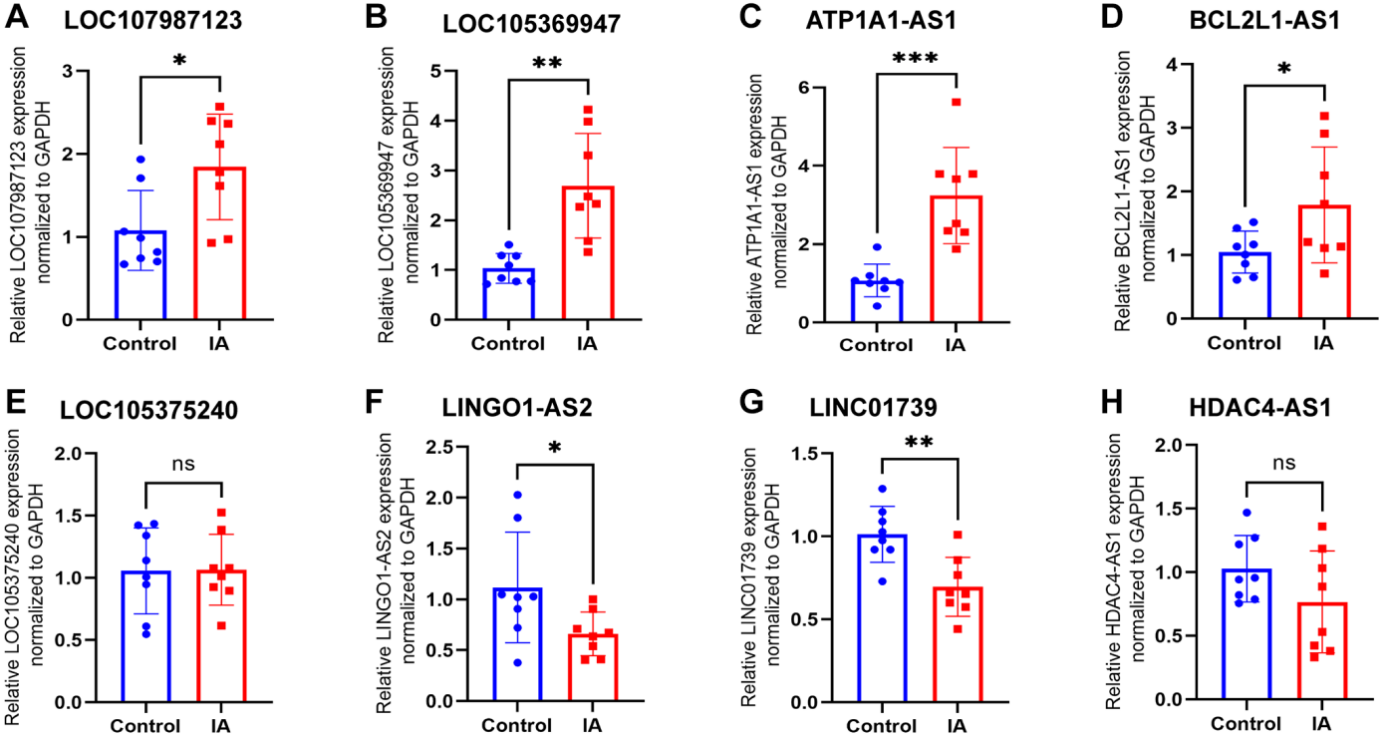
**Expression levels of eight LncRNAs in control and IA plasm exosome identified using qRT-PCR.** (**A**) LOC107987123, (**B**) LOC105369947, (**C**) ATP1A1-AS1, (**D**) BCL2L1-AS1, (**E**) LOC105375240, (**F**) LINGO1-AS2, (**G**) LINC01739, and (**H**) HDAC4-AS1. Data are presented as mean ± standard deviation. All data were analysed using unpaired Student’s *t*-test. An asterisk denotes a significant difference between two groups (^*^p < 0.05, ^**^p < 0.01, ^***^p < 0.001).

To validate the sequencing results and identify LncRNAs with notable differential expression in subsequent functional investigations, four upregulated and four downregulated LncRNAs were selected for qPCR validation (Table 1). The findings demonstrated congruence between the relative expression levels of LOC107987123 (Figure 4A), LOC105369947 (Figure 4B), ATP1A1-AS1 (Figure 4C), BCL2L1-AS1 (Figure 4D), LINGO1-AS2 (Figure 4F), and LINC01739 (Figure 4G) and the outcomes of the sequencing analysis. However, the expression of LOC105375240 (Figure 4E) and HDAC4-AS1 (Figure 4H) in the plasma exosomes of patients with aneurysms was not significantly different (*P* > 0.05) when compared to that in the control group. ATP1A1-AS1 exhibited the most significant difference in expression between the two groups, leading to its selection for further functional experiments. ATP1A1-AS1 functions as a natural antisense counterpart to the Na/K-ATPase α1 gene, located at 1p13.1, and it is expressed in various human tissues [28].

**Table 1:** LncRNAs significantly altered in IA patients as identified through high-throughput sequencing.

**ID**	**Gene name**	**log2FC**	***p*-value**	**Regulated**
XR_001746920.1	LOC107987123	8.158	1.10E-06	Up
XR_945286.2	LOC105369947	7.759	2.21E-05	Up
NR_024126.1	ATP1A1-AS1	6.933	1.34E-03	Up
XR_001754576.1	BCL2L1-AS1	6.899	1.79E-03	Up
NR_135576.1	HDAC4-AS1	−5.033	4.47E-03	Down
NR_146607.1	LINC01739	−5.214	3.80E-03	Down
NR_120362.1	LINGO1-AS2	−5.230	3.13E-03	Down
XR_927189.3	LOC105375240	−6.034	2.46E-03	Down

### Prediction of LncRNAs target genes and competing endogenous RNA (ceRNA) network

**Figure 5:**
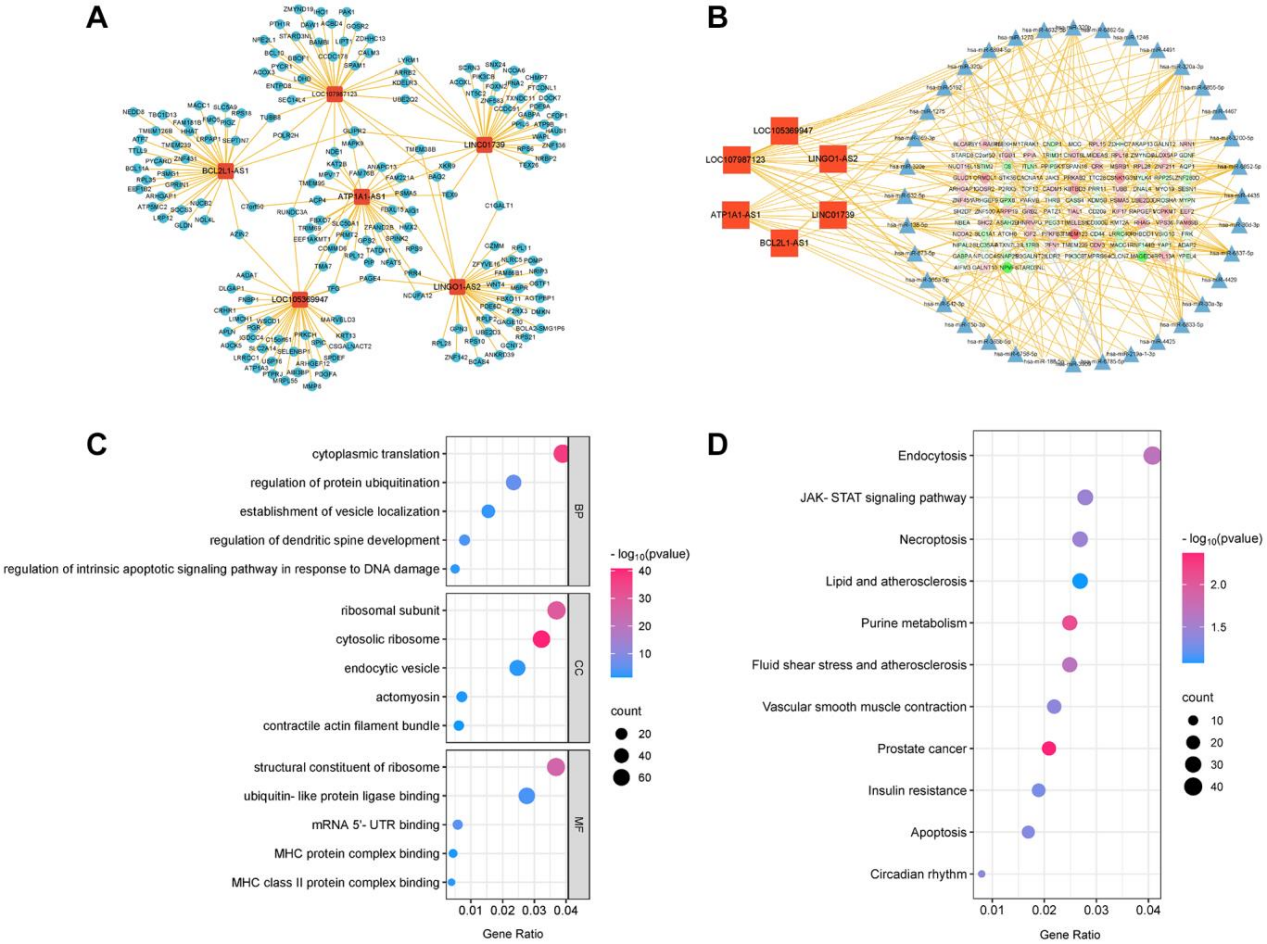
**The LncRNA-mRNA network and LncRNA-microRNA-mRNA network of six key LncRNAs.** (**A**) LncRNA-mRNA network. (**B**) LncRNA-microRNA-mRNA network. (**C**) GO bubble plot of six key LncRNAs target genes. (**D**) KEGG bubble plot of six key LncRNAs target genes.

LncRNAs can function both during and after transcription, in cis-regulatory or trans-regulatory manner. Additionally, they can act as sponges to competitively bind certain microRNAs (miRNAs), thereby regulating miRNA stability and preventing their binding to target genes. To explore the involvement of LncRNAs in aneurysm formation, we identified six key LncRNAs that exhibited differential expression, which was validated by qRT-PCR. Subsequently, we established an interaction network linking these LncRNAs to their corresponding target genes (Figure 5A). The results revealed interactions between the six key LncRNAs and 178 target genes. We created ceRNA networks (Figure 5B) based on the sequencing data of LncRNAs, miRNAs, and mRNA expression profiles. These findings revealed that the six key LncRNAs may directly interact with 36 miRNAs to regulate the functions of 125 genes through the ceRNA mechanism. We performed GO (Figure 5C) and KEGG enrichment analyses (Figure 5D). The results revealed that these target genes are engaged in ‘regulation of intrinsic apoptotic signalling pathway in response to DNA damage’, ‘endocytic vesicle’, and ‘MHC protein complex binding’. Additionally, they are closely linked to the ‘lipid and atherosclerosis’, ‘apoptosis’, and ‘vascular smooth muscle contraction’ pathway. Based on these observations, we hypothesised that these six key LncRNAs contribute to the formation of IAs through the aforementioned pathways.

### Overexpression of ATP1A1-AS1 promotes VSMCs phenotype switching and apoptosis

**Figure 6:**
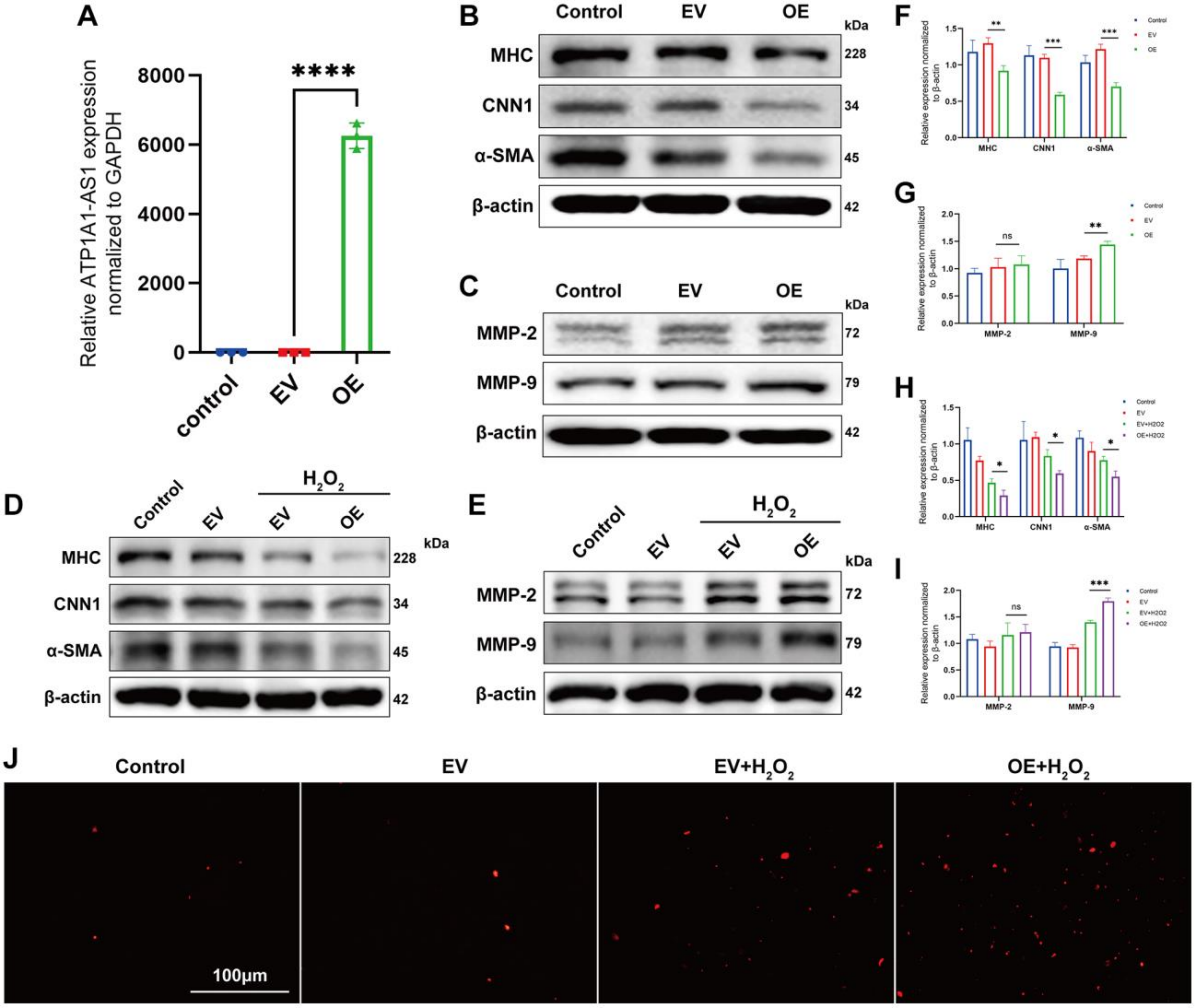
**Overexpression of ATP1A1-AS1 promotes VSMC phenotype switching and apoptosis.** (**A**) ATP1A1-AS1 mimics were used to overexpress ATP1A1-AS1 in VSMCs. The quantification of ATP1A1-AS1 expression was performed using qRT-PCR. (**B**, **C**) WB were performed to quantify the protein expression levels of contractile markers (**B**) and MMP-2 and MMP-9 (**C**) after overexpression of ATP1A1-AS1 in VSMCs. (**D**, **E**) WB was performed to detect contraction markers (**D**) and MMP-2 and MMP-9. (**E**) Protein expression levels after overexpression of ATP1A1-AS1 in pathological VSMCs, simulated using 400 μM hydrogen peroxide. (**F**–**I**) Quantification of protein expression levels. (**J**) TUNEL assay used to detect apoptosis after overexpression of ATP1A1-AS1 in pathological VSMCs. (^*^p < 0.05, ^**^p < 0.01, ^***^p < 0.001).

Given that ATP1A1-AS1 is highly expressed in aneurysmal plasma exosomes and that qRT-PCR results showed significant differences, we conducted a series of functional experiments to investigate its potential role in IA development. Phenotype switching of VSMCs and the activation of matrix metalloproteinases (MMP) are closely associated with aneurysm development [29, 30]. Initially, we constructed an overexpression plasmid of ATP1A1-AS1 and transfected it into VSMCs. The expression of ATP1A1-AS1 significantly increased in VSMCs, 24 h after transfection (Figure 6A). WB revealed a decrease in the expression levels of contractile marker proteins (MYH11, CNN1, and α-SMA) (Figure 6B, 6F) in VSMCs. Furthermore, MMP-9 (Figure 6C, 6G) expression was upregulated after ATP1A1-AS1 overexpression, whereas MMP-2 (Figure 6C, 6G) expression showed no significant change. It is well known that oxidative stress leads to the deterioration of IAs and plays an important role in VSMCs phenotype switching and apoptosis [31]. Therefore, we performed *in vitro* cytopathological simulations using hydrogen peroxide. We exposed VSMCs to hydrogen peroxide (400 μM), and the overexpression of ATP1A1-AS1 led to further reduction in contractile-type marker protein expression (Figure 6D, 6H) and an additional increase in MMP-9 (Figure 6E, 6I) expression. Additionally, ATP1A1-AS1 overexpression increased the apoptotic rate of VSMCs, as observed in the TUNEL assay (Figure 6J). In conclusion, these results indicate that ATP1A1-AS1 may play a role in the development of IAs by promoting the phenotype switching of VSMCs and inducing apoptosis.

### Knockdown of ATP1A1-AS1 inhibits the H_2_O_2_-induced VSMCs phenotype switching and apoptosis

**Figure 7:**
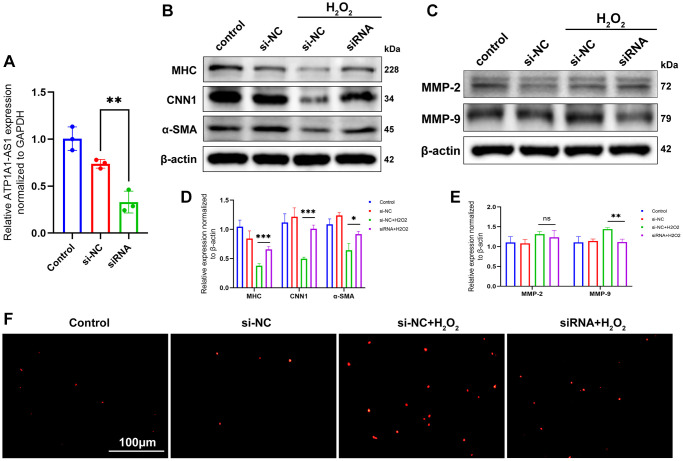
**Knockdown of ATP1A1-AS1 inhibits VSMC phenotype switching and apoptosis.** (**A**) ATP1A1-AS1 siRNA was used to knockdown ATP1A1-AS1 in VSMCs. The expression level of ATP1A1-AS1 was determined using qRT-PCR. (**B**, **C**) WB was performed to detect contraction markers proteins (**B**) and MMP-2 and MMP-9 (**C**) expression levels after knockdown of ATP1A1-AS1 in pathological VSMCs, simulated using 400 μM hydrogen peroxide. (**D**, **E**) Quantification of protein expression levels. (**F**) TUNEL assay used to detect apoptosis after knockdown of ATP1A1-AS1 in pathological VSMCs (^*^p < 0.05, ^**^p < 0.01, ^***^p < 0.001).

The regulatory effect of ATP1A1-AS1 on VSMC was confirmed using ATP1A1-AS1 knockdown. We constructed an siRNA targeting ATP1A1-AS1 and transfected it into VSMCs. qRT-PCR results obtained 24 h after transfection showed successful knockdown of ATP1A1-AS1 (Figure 7A). Compared to VSMCs treated with hydrogen peroxide, ATP1A1-AS1-knockdown VSMCs exhibited elevated expression levels of contractile marker proteins (Figure 7B, 7D) and decreased expression levels of MMP-9 (Figure 7C, 7E), as well as showed no significant changes in the expression of MMP-2. The TUNEL assay showed that the apoptotic rate of VSMCs was reduced following ATP1A1-AS1-specific knockdown (Figure 7F). These findings illustrate that ATP1A1-AS1 knockdown inhibits phenotypic switching and apoptosis of VSMCs, thereby further confirming the regulatory impact of ATP1A1-AS1 on VSMCs.

### Expression of exosomal ATP1A1-AS1 as a novel biomarker for IA

**Figure 8:**
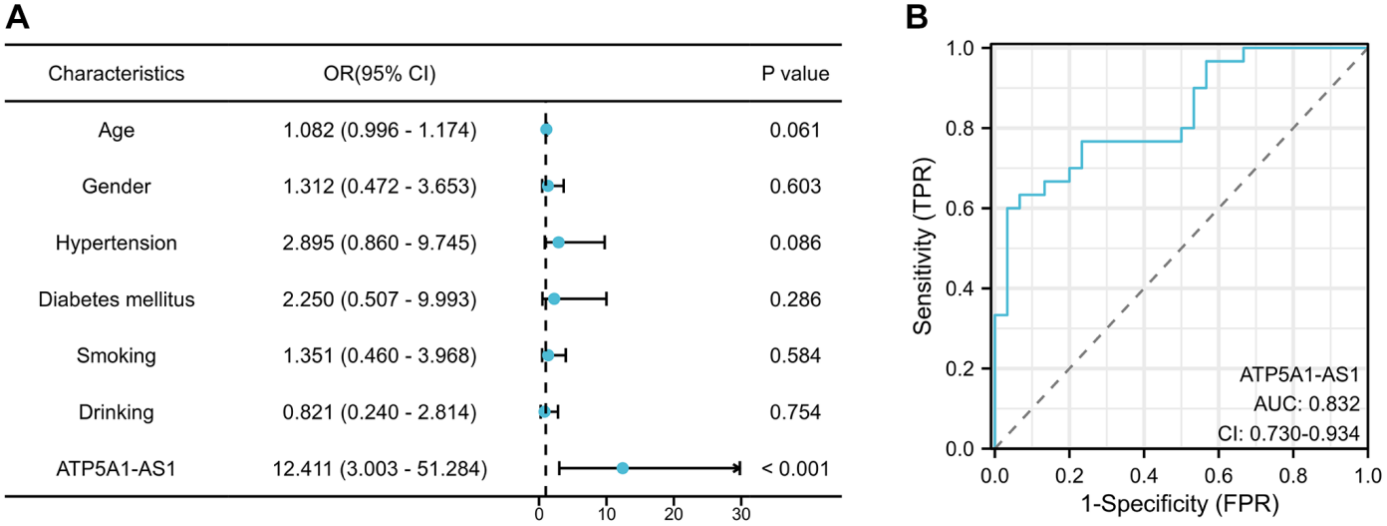
**Expression of exosomal ATP1A1-AS1 as a novel biomarker for IA.** (**A**) Multivariate logistic regression analysis of plasma exosomal ATP1A1-AS1 expression levels in IA patients. (**B**) ROC curves of exosomal ATP1A1-AS1 expression.

To determine whether ATP1A1-AS1 could serve as a clinical biomarker for IA, we examined the expression of ATP1A1-AS1 in the plasma exosomes of our validation cohort. Table 2 shows the clinical characteristics of the validation cohort. We generated receiver operating characteristic (ROC) curve (Figure 8B) to evaluate the diagnostic potential of plasma exosomal ATP1A1-AS1 as a biomarker for IAs. The results revealed that the area under the curve (AUC) of ATP1A1 was 0.832 (95% confidence interval (CI), 0.730–0.934), which helped in distinguishing patients with aneurysms from healthy controls. This implied that the expression level of plasma exosomal ATP1A1-AS1 demonstrated robust diagnostic efficacy. To eliminate interference from other factors, we collected clinical data from patients and performed a multifactorial logistic regression analysis (Figure 8A). The analysis revealed that ATP1A1-AS1 was an independent risk factor for the occurrence of IAs (*p* < 0.001).

**Table 2:** Clinical characteristics of patients with aneurysms and healthy controls in the validation cohort.

	**IA**	**Control**
Mean age (years)	61.6	58.3
Gender		
Male	12	14
Female	18	16
Hypertension		
Yes	11	5
No	19	25
Diabetes mellitus		
Yes	6	3
No	24	27
Drinking		
Yes	7	6
No	23	24
Smoking		
Yes	9	11
No	21	19

## DISCUSSION

In order to investigate the expression profile of LncRNAs in circulating exosomes of IA patients, plasma exosomes from both IA patients and healthy controls were isolated using high-speed centrifugation. Subsequently, whole transcriptome sequencing was performed using NGS. The results revealed the differential expression of 1303 LncRNAs between the two groups, comprising 970 up-regulated and 333 down-regulated. Subsequently, we conducted target gene prediction for differentially expressed LncRNAs and performed functional enrichment analysis on the identified target genes. The results revealed a close association with pathways such as ‘lipid metabolism and atherosclerosis’ and ‘apoptosis’. In the subsequent qRT-PCR validation, ATP1A1-AS1 exhibited the highest differential expression. Further *in vitro* functional experiments revealed that ATP1A1-AS1 can promote VSMCs phenotype switching, enhance MMP9 expression, and induce apoptosis. These findings suggest its potential involvement in influencing the onset of IAs through the aforementioned pathways. Furthermore, the ROC curve demonstrates that the expression levels of plasma exosomal ATP1A1-AS1 exhibit favorable diagnostic efficacy for IAs. This suggests its potential role as a clinical therapeutic target and diagnostic indicator for IAs.

Previous studies have reported that LncRNA ATP1A1-AS1 suppresses the proliferation of thyroid cancer cells and enhances apoptosis by modulating the miR-620–IRF2BP2 axis [32]. Apoptosis is also closely associated with IAs [33]. Hence, we hypothesised that ATP1A1-AS1 promotes IA formation by inducing apoptosis in VSMCs. This was confirmed by TUNEL experiments, which demonstrated that ATP1A1-AS1 promoted apoptosis in VSMCs. Moreover, the phenotypic switching of VSMCs and activation of MMPs are closely linked to aneurysm development [29–31]. In this study, we demonstrated that ATP1A1-AS1 overexpression induced VSMCs phenotype switching and elevated MMP-9 expression. Conversely, the knockdown of ATP1A1-AS1 had the opposite effect. These findings strongly imply that ATP1A1-AS1 contributes to the formation of IAs by promoting VSMCs phenotypic switching and increasing MMP-9 expression.

Finally, we discussed the potential utility of plasma exosomal ATP1A1-AS1 as a diagnostic marker for IAs. Numerous studies have reported the utility of blood ncRNAs as biomarkers for disease diagnosis [34, 35]. Nonetheless, a substantial portion of circulating ncRNAs is passively released from apoptotic and necrotic cells, potentially providing an incomplete representation of the biological alterations within vascular lesions [36]. Conversely, exosomes are actively released into the bloodstream by diverse cell types, encompassing endothelial cells, smooth muscle cells, and more [37]. These exosomes transport ncRNAs, which exert both cardioprotective and reparative effects. Therefore, exosomal ncRNAs may genuinely represent specific molecular biomarkers, in contrast to cell-free ncRNAs [38]. In addition, the protective effects of exosomal membranes may reduce the enzymatic degradation of molecules in body fluids, thereby significantly enhancing the stability of exosomal cargo expression [39]. Niu et al. [40] demonstrated that circulating exosomal miRNAs, as novel biomarkers, exhibit superior diagnostic efficacy compared with plasma miRNAs in large-artery atherosclerotic stroke. In our study, plasma exosomal ATP1A1-AS1 exhibited high sensitivity and specificity as a diagnostic marker, with an impressive AUC value of 0.832. This implies that ATP1A1-AS1 holds promise as a potential biomarker of IAs, thereby enhancing the sensitivity of IAs diagnosis.

Our study had some limitations. The pathological features of intracranial aneurysms include the disruption of the internal elastic lamina, loss of VSMCs, and inflammatory responses within the vascular wall [41]. In this study, our focus was solely on VSMCs, and we did not include endothelial cells and inflammatory cells within the scope of our investigation. Although this study confirmed the effect of ATP1A1-AS1 on VSMCs function *in vitro*, animal experiments are lacking to provide direct evidence of ATP1A1-AS1 affecting IAs formation. Moreover, we did not further explore the regulatory mechanisms by which ATP1A1-AS1 regulates VSMCs function. The current study has shown that LncRNAs can affect disease occurrence by regulating miRNAs or by directly binding to proteins. The ceRNA pathway constructed in this study, along with the predicted target genes, may offer insights for exploring downstream mechanisms. Furthermore, due to the limited sample size and potential factors such as ethnic variations, additional validation of ATP1A1-AS1's diagnostic effectiveness should be conducted in independent prospective IA cohorts.

In summary, our study comprehensively characterised the expression of plasma exosomal LncRNAs in patients with IAs and identified specific DE LncRNAs. We discovered that ATP1A1-AS1 induces phenotype switching, promotes apoptosis, and upregulates MMP-9 expression in VSMCs, potentially contributing to IA formation. Furthermore, plasma exosomal ATP1A1-AS1 has strong potential as a clinical diagnostic marker for IAs. These findings emphasise the crucial role of exosomal LncRNAs in IAs and validate their potential use as biomarkers and therapeutic targets.

## MATERIALS AND METHODS

### Patients and ethics

Thirty patients with IAs diagnosed by CTA or DSA and thirty healthy volunteers who underwent CTA to exclude IAs were recruited by the Department of Neurosurgery of The Affiliated Hospital of Qingdao University. The discovery cohort, comprised 4 patients and 4 healthy volunteers, and 30 patients and 30 healthy volunteers were designated as the validation cohort. In the discovery cohort, we examined the plasma exosomal LncRNAs using NGS. Next, we confirmed the distinct expression profiles of plasma exosomal LncRNAs in the LncRNA candidates in the validation cohort using qRT-PCR.

The eligibility criteria for the IA patients were as follows: (1) patients with IAs diagnosed by CTA or DSA, ruptured or unruptured; (2) older than 18 years; and (3) with complete clinical data. The eligibility criteria for the control group were as follows: (1) healthy volunteers who underwent CTA to exclude IAs and (2) age-matched patients (aged 45–75 years) with aneurysms. The exclusion criteria were as follows: (1) previous brain tumours or other cerebrovascular diseases, (2) family history of neurological or tumour-related diseases, and (3) other serious underlying diseases.

### Exosome isolation and identification

Plasma samples obtained from IA patients and healthy individuals were quickly thawed at 37°C and then underwent centrifugation at 2,000 × g, 4°C, for 30 min to separate cellular debris. The resulting supernatant underwent centrifugation at 10,000 × g, 4°C, for 45 min to eliminate larger vesicles. The supernatant was carefully removed and filtered through a 0.45μm filter membrane to collect the filtrate. Subsequently, the supernatant was aspirated and pellets were reconstituted in 10 ml of 1× PBS. The resuspended pellet was then subjected to another round of centrifugation at 4°C for 70 min at 100,000 × g. Lastly, the pellet was re-suspended in PBS and characterised using NTA (NanoFCM N30E, China) to determine the exosome diameter. The morphological features of the exosomes were assessed by transmission electron microscopy (TEM) analysis (Hitachi HT-7700, Japan). Exosome protein markers (CD9, CD81, and TSG101) were determined using WB.

### Next-generation sequencing

Extraction of exosomal RNA was performed using the Magzol Reagent (Magen, China). The RNA yield was quantified using Qubit (Thermo Fisher Scientific, USA) and Agilent 2200 TapeStation (Agilent Technologies, USA). Briefly, rRNA molecules were selectively removed from the exosomal RNA pool using the QIAseq FastSelect RNA Removal Kit (QIAGEN, Germany) and subsequently fragmented into approximately 200-base pair fragments. The resulting RNA fragments underwent a two-step cDNA synthesis process, encompassing first strand and second strand cDNA synthesis, followed by adaptor ligation and enrichment with a reduced number of amplification cycles, adhering to the protocols outlined in the NEBNext^®^ Ultra™ RNA LibraryPrep Kit for Illumina (NEB, USA). Quality assessment of the purified library products was performed using Agilent 2200 TapeStation and Qubit. Subsequently, the libraries were sequenced on an Illumina platform (Illumina, USA) with a paired-end configuration of 150 bp (RiboBio Co., Ltd., China).

### Quality control and identification of new LncRNAs

Raw FASTQ sequences were processed to screen trailing sequences with Phred quality scores below 20 using Trimmomatic (v0.36) employing the following parameters: TRAILING, 20; SLIDINGWINDOW, 4:15; and MINLEN, 52. This ensured uniform sequence lengths throughout the dataset. Subsequently, raw data were subjected to an initial filtering process to exclude low-quality reads. High-quality filtered data were then subjected to assembly using StringTie software, leveraging reads aligned to the reference genome. The resulting transcripts were annotated using GFF Compare. To identify potential LncRNAs, transcripts with unknown functions were scrutinised. Potential protein-coding RNAs were differentiated from non-coding RNAs by applying stringent criteria, including minimum length and exon number thresholds. Transcripts exceeding 200 nucleotides in length and exhibiting a predicted open reading frame (ORF) shorter than 300 nucleotides were retained as candidate LncRNAs. To further refine the selection and classification, the candidates underwent processes involving the CPC, CNCI, and Pfam tools.

### Differential expression analysis

Significant differential gene expression was determined by applying stringent criteria: an adjusted *P*-value threshold of <0.05, and a | log2(fold change) | >1.5, using the DESeq/DESeq2/edgeR/DEGseq software. Following this, a hierarchical clustering analysis was performed using the ‘gplots’ package in R. The clustering was based on the transcripts per million (TPM) values of the differential expression (DE) genes across various groups. Colours were used to visually represent distinct clustering information, illustrating similarities in expression patterns within the same group, indicative of shared functions or participation in common biological processes.

### GO terms and KEGG pathway enrichment analysis

To reveal the Gene Ontology (GO) annotations and pathways enriched by the differentially expressed genes, we conducted GO term and Kyoto Encyclopedia of Genes and Genomes (KEGG) pathway enrichment analyses. These analyses were executed utilising the ‘clusterProfiler’ package within the R Bioconductor framework. Within the scope of this investigation, the ‘clusterProfiler’ package was used to identify and visually represent the enriched GO terms and KEGG pathways associated with all differentially expressed genes.

### qRT-PCR

Total exosomal RNA was extracted using TRIZOL reagent (Bioflux, China). The extracted RNA was reverse transcribed into cDNA using SPARK script II RT kit (With gDNA Eraser) (Shandong Sparkjade Biotechnology Co., Ltd., China). LncRNA expression levels were quantified using qRT-PCR with SYBR Green qPCR Mix (Yeasen, China) and a LightCycler 96 instrument (Roche, Switzerland). To normalise LncRNA expression, Glyceraldehyde-3-phosphate dehydrogenase (GAPDH) was used as an internal reference. The data were obtained from three independent experiments and then averaged using the 2^−ΔΔCT^ method. The primer sequences are listed in Table 3.

**Table 3:** Primer sequences for qRT-PCR and siRNA sequence.

**Primer name**	**Primer sequences (5′–3′)**
GAPDH(HU)-F	AAGAAGGTGGTGAAGCAGGC
GAPDH(HU)-R	TCCACCACCCAGTTGCTGTA
LOC107987123-F	TTGCCATCTGCGTCTTTGTAAT
LOC107987123-R	ACATGATCCACCTCTTGGGAGT
LOC105369947-F	CAAGGGCTACAAAGGTGAATGA
LOC105369947-R	GTTGGTTGTTAAGGCAGGATGA
ATP1A1-AS1-F	GCCTCCTTGCCTGTGAGATG
ATP1A1-AS1-R	CAAATGCACGATTTCACTCGG
BCL2L1-AS1-F	CCACCCAAAGCTATGGCGAC
BCL2L1-AS1-R	TCTGCAAATCAGCGATGGAACT
LOC105375240-F	GTGAGTTGTGAGCGAGGCAGAT
LOC105375240-R	AGACTACGGTGGAAATGGAAGGT
LINGO1-AS2-F	TCCTTGTCCCAGCTCCACTCAC
LINGO1-AS2-R	GACCCATCCACTTCAGGCTTCC
LINC01739-F	GGCCAATTTCTCCCAAGATGTG
LINC01739-R	CTTCCAGACGCTGAGTCGCTGT
HDAC4-AS1-F	CACTGTCCCCGGAATCCCGCGTTCC
HDAC4-AS1-R	AATGGCCTACAATCGAAGCGAACG
si-ATP1A1-AS1-sense	GUGGUUUCCAAACUUGAAUTT
si-ATP1A1-AS1-antisense	AUUCAAGUUUGGAAACCACTT

### Cell culture and transfection

Human brain vascular smooth muscle cells (HBVSMCs) were cultivated under controlled conditions. Specifically, they were grown in a humidified atmosphere at 37°C with 5% carbon dioxide, in Dulbecco’s modified eagle’s medium (DMEM) enriched with 10% fetal bovine serum (Inner Mongolia Eppen Biotechnology Co., Ltd., China) and supplemented with streptomycin (50 mg/mL) and penicillin (50 U/mL). A customised siRNA sequence was designed and synthesised (GenePharma, Shanghai, China) to specifically target ATP1A1-AS1. To achieve knockdown of ATP1A1-AS1 in VSMCs, stable transfection with siRNA was conducted, while a non-silencing siRNA served as the control, following the RNAi protocol provided by the manufacturer. The detailed sequences of the siRNA oligonucleotides are shown in Table 3. The ATP1A1-AS1 sequence was synthesised and subcloned into pcDNA3.1 vector (GenePharma, Shanghai, China). To establish the ectopic expression of ATP1A1-AS1 in VSMCs, stable transfection with pcDNA3.1-ATP1A1-AS1 was carried out, with VSMCs transfected with an empty pcDNA3.1 vector serving as the control.

### Western blot

Treated cells were lysed for 30 min on ice in RIPA buffer containing a protease inhibitor cocktail (Meilun, Dalian, China). Equal protein loading was ensured by measuring the protein concentration in each sample using a BCA protein assay kit (Meilun, Dalian, China). Loading buffer was added to the protein, and the mixture was heated at 98°C for 10 min. Subsequently, the samples were separated by 10% SDS polyacrylamide gel electrophoresis (SDS-PAGE) and transferred onto polyvinylidene fluoride (PVDF) membranes. The membranes were then blocked with protein-free rapid blocking buffer (Meilun, Dalian, China) for 10 minutes, and probed with the following primary antibodies: anti-α-SMA (BM0002, Boster, Wuhan, China), anti-CNN1 (abs171608, Absin, Shanghai, China), anti-MYH11 (K002095P, Solarbio, Beijing, China), anti-MMP2 (A00286-2, Boster, Wuhan, China), and anti-MMP9 antibodies (BM4089, Boster, Wuhan, China). After three washes, the membranes were incubated with HRP peroxidase-conjugated AffiniPure goat anti-rabbit IgG (BA1056, Boster, Wuhan, China) for 1 h. The signals were detected and visualised using a chemiluminescence imaging system (Millipore, Billerica, MA, USA). Subsequently, the protein levels were quantified and assessed using ImageJ software and subsequently normalised based on the corresponding β-actin levels.

### Apoptosis assay

Apoptosis was assessed using a TUNEL Apoptosis Assay Kit (Abbkine, Beijing, China). Cells were cultured in 96-well plates and treated to induce apoptosis. Subsequently, cells were fixed with 4% paraformaldehyde for 30 min. Following fixation, three PBS washes were performed, and the cells were permeabilised with 0.3% Triton X-100 for 30 min. After additional washes, a TdT reaction buffer was prepared and incubated with the cells for 1 h at 37°C, protected from light. The cells were re-stained with DAPI after three washes, followed by another round of washing with PBS. Finally, the cells were observed by fluorescence microscopy at excitation/emission wavelengths set at 555 nm/565 nm.

### Statistical analysis

Statistical analyses were performed using SPSS version 27.0 (SPSS Incorporated), and graphical representations were created using GraphPad Prism 9.0. Data comparisons were conducted using unpaired *t*-tests or Mann-Whitney *U*-tests. Receiver operating characteristic (ROC) curves were constructed, and the area under the curve (AUC) was calculated to evaluate the diagnostic potential of LncRNAs. To ascertain the correlation between plasma exosomal LncRNA expression levels and their diagnostic relevance for IA, multivariate logistic regression analyses were performed. Statistical significance was set at *p* < 0.05. Each set of experimental data was generated from a minimum of three independent replicates.
